# Clinical and Histologic Remission in an Adult Crohn’s Disease Patient Following the Specific Carbohydrate Diet and Its Impact on Healthcare Costs

**DOI:** 10.7759/cureus.22032

**Published:** 2022-02-08

**Authors:** Ali Arjomand, David L Suskind

**Affiliations:** 1 Nutrition Clinic, Modulla Health, Bellevue, USA; 2 Pediatric Gastroenterology, Seattle Children’s Hospital, Seattle, USA; 3 Department of Pediatrics, University of Washington, Seattle, USA

**Keywords:** dietary modification, value based healthcare, low cost healthcare, histologic remission, nutrition, adjunct therapy, specific carbohydrate diet, crohn's disease, inflammatory bowel disease

## Abstract

Crohn’s disease (CD) is an immune-mediated inflammatory disorder of the gastrointestinal tract. While the etiology is not fully elucidated, the intestinal microbiome is believed to initiate and maintain immune activation in CD. The intestinal microbiome is highly responsive to its environment, including host dietary patterns. As such, dietary interventions have the potential to modulate intestinal microbiome composition and function and improve disease outcomes. We present a retrospective chart review of an adult male with complicated Crohn’s disease who was non-responsive to medical management. The patient began the specific carbohydrate diet (SCD) in February 2017 and maintained it for 42 months. The patient tolerated the SCD well and has been asymptomatic for 40 months on the SCD. Stool fecal calprotectin (FCP) decreased from 493 ug/g at baseline to 70 ug/g at month three and remained in the normal range thereafter. Endoscopy with biopsy at month 12 showed normal histology in the colon and terminal ileum. Magnetic resonance enterography (MRE) showed resolution of prior jejunal inflammation. Inflammatory bowel disease (IBD) associated healthcare costs were $42,688 in the 12 months preceding the intervention and $2,797/year with SCD. This represented a 94% reduction in healthcare insurance costs and a 91% reduction in out-of-pocket patient expenses. This case highlights the rapid and sustainable benefits of the SCD intervention in Crohn’s disease from both a clinical and economic standpoint.

## Introduction

Crohn’s disease (CD) is an immune-mediated inflammatory disorder of the gastrointestinal tract. While the etiology has not been fully elucidated, the CD is thought to be a dysregulation of immune system activation and upregulation triggered by environmental factors and altered intestinal microbiome ecology. Despite the role of the environment, current inflammatory bowel disease (IBD) therapeutic approaches target immunologic response pathways with little regard to primary driving factors. Increasing evidence points to an altered intestinal microbiome composition as a key determinant of gastrointestinal health and disease [1]. In the case of IBD, altered intestinal microbiome composition has been shown to degrade mucosal barrier integrity, increase intestinal permeability, and in combination, may be involved in the etiology and/or progression of the disease [2].

The primary focus of IBD therapy is to suppress the immune system’s ability to activate and sustain an inflammatory response. While this approach can be effective, it does not reliably induce remission in as many as 55% of patients, and 23% to 46% of those that respond lose response over time [3]. Given the incomplete efficacy of medication therapy and accompanying high cost, interest in the role of environmental factors in disease activity has risen in recent years.

Diet is perhaps the most dominant environmental factor in IBD. It also represents environmental exposure that can be modulated by patients to drive better outcomes. This notion is further reinforced in IBD where exposure occurs at the intestinal-environment interface, a process intrinsically coupled with nutritional status, intestinal microbiome, mucosal integrity, and inflammatory response, all hallmarks of IBD pathophysiology [4,5]. As such, dietary modification offers a unique point of intervention and a potential mechanistic target that can be implemented in conjunction with current medication-based strategies to extend currently attainable outcomes.

To date, the most widely studied whole-food intervention in IBD is the specific carbohydrate diet (SCD). SCD was initially created in 1924 to treat celiac disease [6]. It was later used for the treatment of IBD [7]. The SCD eliminates all grains, most starches, sugar (except honey), dairy products (except butter, 24-hour fermented yogurt and hard cheeses, which are essentially lactose-free), and most store-bought, processed, or prepackaged foods. Preliminary studies of SCD intervention in pediatric and adult IBD patients have demonstrated promising results in low-number patient cohorts, including achieving clinical and mucosal remission and normalization of blood and stool laboratory markers [8-14]. A recent randomized trial in adult Crohn’s disease patients demonstrated symptomatic remission and fecal calprotectin (FCP) response in 46.5% and 34.6% of patients, respectively, after a six-week regimented SCD intervention, matching the response seen in the Mediterranean diet intervention arm [15].

Reports demonstrating long-term adherence to dietary intervention in IBD, objective assessment of response and associated reduction in healthcare cost have not been reported to date. Herein, we present the case report of an adult patient with intractable Crohn’s disease non-responsive to medical management who demonstrated endoscopic, radiologic, histologic, and biochemical responses to the SCD intervention. We furthermore report the healthcare cost savings over a 42-month SCD intervention period.

## Case presentation

A 54-year-old male initially presented with abdominal pain and diarrhea at age 35. Initial evaluation by small bowel follow-through revealed multiple segments of luminal narrowing within the small bowel. Therapy was initiated with mercaptopurine (1.5 mg/kg PO qHS for 12 months). The patient subsequently experienced two episodes of partial small bowel obstruction in the following six months, requiring treatment escalation to infliximab in conjunction with prednisone (40 mg/day). He developed a third partial small bowel obstruction and underwent scheduled laparoscopic resection of 50 cm of fibrostenotic terminal ileum. He was maintained on infliximab (5 mg/kg/dose qHS 8 weeks) and azathioprine. The patient remained in clinical remission for three years.

The patient became symptomatic again at 40 years of age with watery diarrhea (2-8 BM/day), weight loss, and bilateral lower extremity edema. His C-reactive protein (CRP) was 39.4 mg/L and albumin <1.0 g/dL (reference range 3.5-5.2 g/dL). He initiated adalimumab treatment (40 mg every two weeks after induction) in February 2008. The patient’s symptoms continued to worsen over the following five months. He was admitted to the hospital with severe protein energy malnutrition (weight loss of 10.7% over six months). Colonoscopy revealed a terminal ileum with marked linear deep serpiginous ulcers and ileocecal valve edema and narrowing. The biopsy revealed severely chronic active inflammation. Computerized tomography (CT) revealed active distal small bowel inflammation. The patient began intravenous corticosteroids and total parenteral nutrition (TPN). Hospitalization was complicated by a large thrombus at an arterial bifurcation (saddle embolism), leading to ICU transfer and anticoagulation therapy. He was discharged after one month in the hospital and continued at-home TPN for four months. He transitioned from adalimumab to natalizumab (300 mg every four weeks) during the hospitalization, along with a corticosteroid (40 mg/day) for six months. He returned to adalimumab (40 mg every 14 days) due to concerns with natalizumab safety and a lack of alternative therapies in 2008. He additionally tapered the corticosteroid to 20 mg/day. Adalimumab was increased to weekly administration with uninterrupted corticosteroid therapy (10 to 20 mg/day) for the following seven years.

Throughout this period the patient experienced disease flares with abdominal pain and vomiting, resulting in multiple emergency department (ED) visits and hospitalizations. At 47 years of age, he presented to the ED with severe pain and hypotension, resulting in ICU admission. He was diagnosed with *Campylobacter jejuni *sepsis and acute kidney failure. He recovered with antibiotic therapy and was discharged after recovering kidney function.

One year later, he presented with abdominal pain and vomiting. Magnetic resonance enterography (MRE) revealed chronic fibrostenosing disease involving an approximately 4.7 cm segment of the jejunum with an enteroenteric fistula. An additional focus of active Crohn's enteritis was also noted in the distal small bowel. The patient transitioned from adalimumab to vedolizumab (300 mg every eight weeks after induction), corticosteroids (20 mg/day) and budesonide (9 mg/day).

The patient experienced worsening of symptoms over the following six months, along with a 9 kg weight loss. TPN was initiated one month prior to the scheduled surgery. The patient underwent repeat bowel resection (30 cm of ileum with sparing of the ileocecal valve) at 48 years of age. He was discharged after 10 days, successfully reintroduced solid foods, and gained 11 kg in the subsequent six months. He remained on vedolizumab (300 mg every eight weeks) therapy for one year. MRI performed one year after surgery showed fibrostenosing disease involving the terminal and distal ileum extending approximately 14 cm in length. A 3 cm portion of the terminal ileum appeared more fibrotic with active inflammation of the adjacent distal ileum. There was a 4 cm segment of jejunal inflammation within the left upper quadrant with no obstructive change. The patient was offered ustekinumab as a replacement for vedolizumab, which he refused to implement. Instead, the patient decided on his own to initiate SCD intervention. Physician oversight allowed intervention if the patient’s disease worsened or if the patient decompensated. Corticosteroids (40 mg/day) were initiated as a precaution as there was no assurance that SCD would demonstrate efficacy.

Dietary intervention

At the time of dietary intervention, the patient’s diet, as determined by his food diary, consisted of a Western-style diet composed primarily of refined flours (bread and pasta), starches (rice and potatoes), poultry, beef, and packaged foods. He consumed a very limited range of fruits and vegetables, minimized fast-food, and avoided high-residue foods (whole grains, greens, nuts, seeds, and berries).

In February 2017, the patient self-implemented a strict SCD intervention. He prepared SCD-compliant foods at home, guided by his own food tolerances and preferences and several online resources (for example: nimbal.org and ibdnavigator.com). Diet was supplemented with iron (20 mg/day), vitamin B12 (1,000 mcg/day), vitamin D3 (15,000 IU/day), and a daily multivitamin.

Clinical outcome

On day 10 of the SCD intervention, the patient reported to his gastroenterologist loss of abdominal and lower back pain and a 2.2 kg drop in body weight. Over the following weeks, he progressively expanded the range of SCD-compliant ingredients in a methodical and stage-wise manner with the aid of a journal and food diary. The patient reported to his gastroenterologist continued symptom improvement, reduced bowel frequency, and improved stool consistency. The corticosteroid dose was tapered to 20 mg/day by week 6, at which point he was consuming increasing amounts and a wider range of dietary fibers from whole foods, as recorded in a food diary.

At week 12, the patient reported complete loss of all joint pain, one to two partial or fully formed bowel movements per day of large mass, improved energy, sleep, and appetite. The patient tapered off corticosteroids by week 26 and was without medication for the first time since his initial diagnosis in 2001.

The patient progressed to more advanced SCD ingredients, customizing his expansion based on self-observed tolerance, reduced symptoms, and culinary preference. At the time of this report, his diet consisted of a wide range of ingredients, including fruits, vegetables, fresh greens, whole nuts and seeds, fermented foods, some legumes, eggs, seafood, poultry, and beef. Supplementation consisted of iron (20 mg/day), vitamin B12 (1,000 mcg/day), vitamin D3 (15,000 IU/day), and a daily multivitamin.

The patient reported a loss of 6.8 kg of body weight at month 12 of the SCD intervention, equating to a BMI drop from 21.6 to 19.7 kg/m^2^ (normal range, 18.5 to 24.9 kg/m^2^). The patient has maintained the SCD for 42 months at the time of this report.

Laboratory, endoscopic, radiologic, and histologic assessments

**Figure 1:**
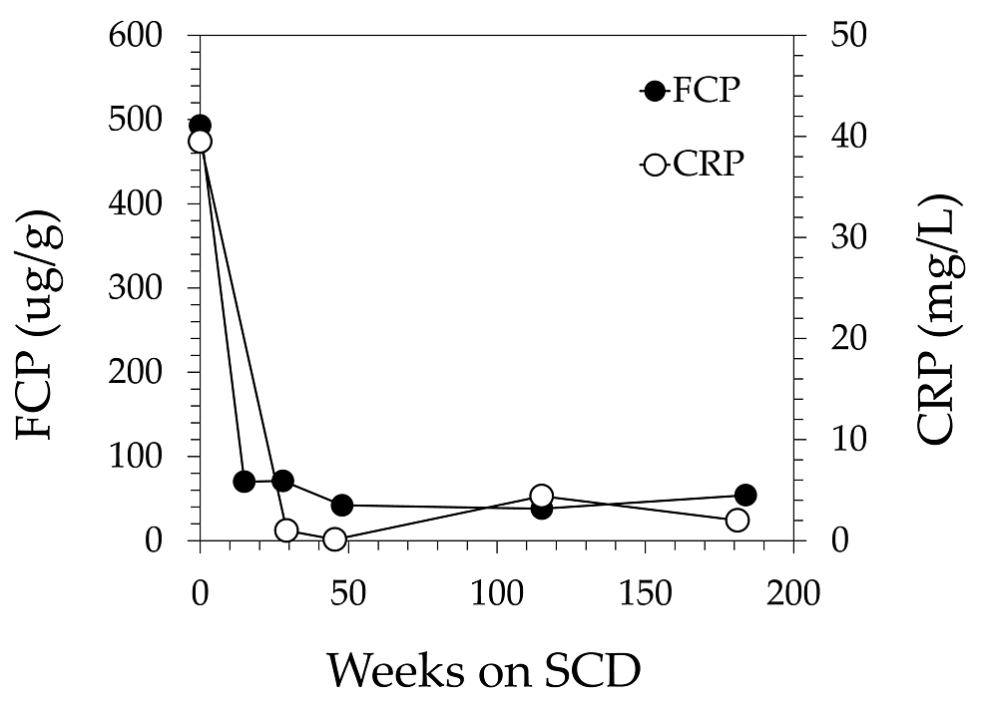
Inflammatory markers showed a rapid and sustained response to the SCD intervention over a 184-week period. SCD: specific carbohydrate diet; FCP: fecal calprotectin; CRP: C-reactive protein.

A range of analytes were measured at baseline and after initiation of the SCD, including CRP, fecal calprotectin (FCP), IL-6 and IL-8, and markers of nutritional status. Figure 1 shows CRP and FCP values at baseline and throughout the 184-week SCD intervention. CRP dropped from 39.5 mg/L at baseline to 1 mg/L at week 29 and remained in the normal range throughout the reporting period. The FCP dropped from 493 ug/g at baseline to 70 ug/g at week 15 and remained in the normal range for the remainder of the reporting period.

Additional analytes were measured, including cytokine levels, lipid profile, and nutritional labs (see Table 1). Inflammatory mediators IL-6, IL-8, and tumor necrosis factor alpha (TNF-α) dropped from baseline values of 13.8, 8, and 1.2 pg/mL, respectively, to 2.6, 3.5, and 0.8 pg/mL at week 22. Cholesterol, high-density lipoprotein (HDL), and low-density lipoprotein (LDL) started and remained in the normal range. Vitamin D, 25-OH values dropped from 44.4 ng/mL to 35.6 ng/mL. Markers of iron status, anemia and cardiovascular health remained normal.

**Table 1:** Laboratory measurements demonstrated improved inflammatory, lipid, glucose, cardiovascular and nutritional markers after 22 weeks of SCD. SCD: specific carbohydrate diet; TNF-α: tumor necrosis factor alpha; LDL: low-density lipoprotein; HDL: high-density lipoprotein.

Analyte	Units	Reference range	Baseline	SCD (week 22)
IL-6	pg/mL	-	13.8	2.6
IL-8	pg/mL	-	8.0	3.5
TNF-α	pg/mL	-	1.2	0.8
Cholesterol	mg/dL	<200	114	109
LDL	mg/dL	<100	47	40
HDL	mg/dL	40-100	36	39
Triglycerides	mg/dL	70-150	156	151
Glucose	mg/dL	65-110	82	81
Insulin	uIU/mL	6-35	6.1	3.6
Hgb A1C	%	4-5.9	5.1	4.5
Vitamin D, 25-OH	ng/mL	8-80	44.4	35.6
Methylmalonic acid	nmol/mL	<400	186	282
Ferritin	ng/mL	20-250	70	70
Hematocrit	%	42-52	39.2	37.9
Hemoglobin	g/dL	13-18	12.7	12.6
Albumin	g/dL	3.5-5.5	3.8	3.8
Systolic BP	mm Hg	<120	103	95
Diastolic BP	mm Hg	<80	57	55
Weight	kg	-	70.5	64.1
BMI	kg/m^2^	18.5-24.9	21.6	19.7

Endoscopic, radiologic, and histologic assessments were performed at baseline and month 12 of the SCD (see Table 2). Magnetic resonance enterography (MRE) showed a reduction of fibrostenosing disease involving the terminal and distal ileum from 14 cm at baseline to 5 cm at month 12, along with resolution of a 4 cm segment of jejunal inflammation. Endoscopy showed congestion, edema, erythema, exudates, friability, and ulceration in the terminal ileum at baseline. Endoscopy performed at month 12 showed normal mucosa (biopsy) in the terminal ileum and normal mucosa in the whole colon.

**Table 2:** Imaging and endoscopic reports at baseline and month 12 of the SCD intervention. SCD: specific carbohydrate diet; MRE: magnetic resonance enterography.

	Baseline	SCD month 12
MRE	14 cm of fibrostenosing disease involving the terminal and distal ileum. A 3 cm portion of the terminal ileum appears more fibrotic with active inflammation of the adjacent distal ileum. A 4 cm segment of jejunal inflammation within the left upper quadrant. No obstructive changes.	A 5 cm segment of fibrostenosing disease involving the distal ileum with proximal dilatation. Normal terminal ileal peristalsis. No generalized bowel obstruction. Resolution of prior jejunal inflammation.
Endoscopy	Congestion, edema, erythema, exudates, friability, and ulceration in the terminal ileum are compatible with Crohn’s disease (biopsy).	Normal mucosa in the terminal ileum (biopsy). Normal mucosa in the whole colon. Stricture in the terminal ileum.

Healthcare cost reduction

A complete record of the patient’s healthcare expenses covering the 12-month trailing period prior to the SCD intervention and the 42-month SCD intervention period was compiled and examined. IBD claims were separated from non-IBD claims and reported as either out-of-pocket patient costs (deductibles, copays, and co-insurance) or health plan costs (preferred-provider organization, self-insured by a Fortune 500 company). Indirect costs attributed to lost productivity or the cost of implementing the SCD intervention were not included in the cost analysis.

**Figure 2:**
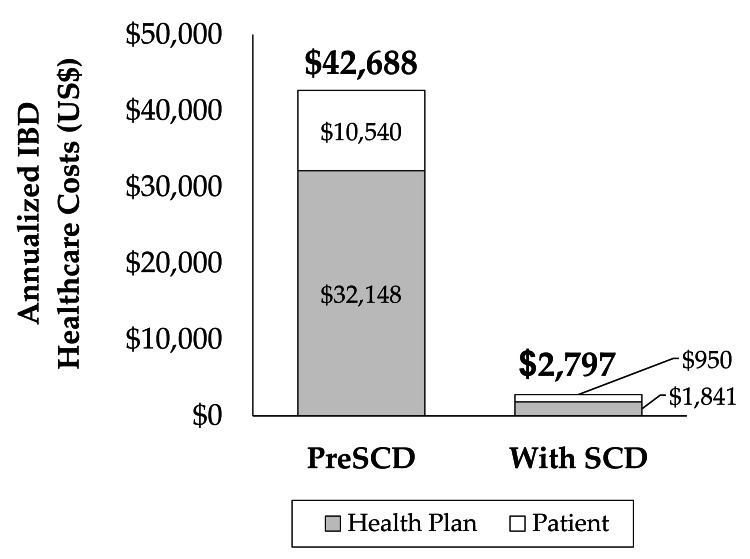
Total direct IBD annual healthcare costs prior to and after introduction of the SCD. IBD: inflammatory bowel disease; SCD: specific carbohydrate diet.

IBD healthcare costs during the 42-month SCD intervention period were annualized to permit comparison with the 12-month preSCD trailing period. Figure 2 shows IBD healthcare costs prior to and during the SCD intervention period. IBD healthcare costs in the 12-month trailing period prior to the SCD intervention were $42,688. The patient’s health plan paid $32,148, and the patient paid $10,540. Annualized IBD healthcare costs during the SCD intervention period were $2,796/year. The patient’s health plan paid $1,841/year, and the patient paid $956/year.

The SCD intervention described in this report reduced health plan costs by 94.3% and patient costs by 90.9%. Notably, the 12 months preceding the SCD intervention were free of emergency department visits, hospitalizations, or surgery, any of which would magnify the healthcare cost reduction noted above. Total IBD healthcare cost savings with SCD intervention over a four-year period are projected at $159,590, comprised of $121,230 health plan savings and $38,335 patient savings.

## Discussion

This case report highlights the positive effects of dietary therapy on inflammatory burden, mucosal healing, and healthcare costs. The prevalence of adults living with IBD in the US was estimated at 1.3% of the population (three million) [16]. This represented an increase from the 0.9% prevalence reported in 1999 (two million adults). Estimates of costs associated with IBD range between $14.6 and $31.6 billion in 2014. As in most chronic conditions, the cost of care for IBD is primarily driven by hospital utilization, surgery, and pharmacotherapies. The cost of IBD management is increasing annually, primarily driven by cost increased pharmacotherapies such as biologic therapies, with pharmacy utilization costs accounting for nearly one-half of all costs [17].

A current Crohn’s and Colitis Foundation initiative to study the cost burden of IBD notes the pressing need for cost-effective strategies to address the cost borne by patients and families affected by IBD. These families incurred greater than the three-fold higher direct cost of care compared with non-IBD controls ($22,987 vs $6,956 per-member per-year paid claims) and more than twice the out-of-pocket costs ($2,213 vs $979 per-year) [18]. A successful and safely delivered dietary intervention could help drive down these costs over the short and long term.

Evidence exists for the potential benefits of integrating medication and dietary therapy. Chiba et al. showed significant improvement in response rate with infliximab with the implementation of a plant-based diet [19].

Dietary interventions, such as the SCD, require significant commitment and lifestyle change. Central to this undertaking is patient education, skill-building, and access to support resources. While the patient described in this report had the means, knowledge and drive to undertake such a lifestyle change, this may not be feasible for all patients. Concerns regarding food-associated quality of life, risk of malnutrition and social acceptance should additionally be addressed in any strict dietary intervention. As with this patient, successful dietary intervention requires tailored implementation, progressive expansion, and nutrient diversification to help avoid the risk of nutrient deficiency while maximizing the likelihood of success. Such dietary interventions should be performed in a coordinated care setting, under professional supervision, with support from a multi-disciplinary care team with experience in the intervention.

There are currently no formal guidelines for dietary intervention from the major gastroenterology societies. A recent report provides recommendations on the consumption or avoidance of food components and additives but does not integrate those findings into a comprehensive dietary plan for Crohn’s disease patients [20]. While more research is still required to define the optimal diet for Crohn’s disease, dietary interventions can have a positive effect on both clinical symptoms and inflammatory burden. Understanding diet’s impact is the proverbial low-hanging fruit and has the potential to improve clinical outcomes for IBD patients as well as help reverse the financial burden of this chronic disease.

This case study has certain limitations. Firstly, as with any case report, it presents outcomes observed in a single patient. As such, interpretation of these results for other patients should be made cautiously and in the context of individual patient status. Secondly, this is a retrospective case presentation and may not reflect cases where patients failed to achieve similar outcomes. This case report has several strengths. Firstly, it represents the real-world outcome of an adult patient with long-lasting refractory Crohn’s disease who was under the care of a community gastroenterology clinic. Secondly, the report focuses on objective and validated markers of response, including biochemical, histologic, and radiologic findings. Thirdly, the 42-month intervention period is longer than reports of dietary intervention published elsewhere, demonstrating sustained adherence and sustained response. Finally, the study is the first report to our knowledge that quantitatively assessed the real-world impact of dietary intervention on direct IBD healthcare costs, demonstrating dramatically reduced costs to both patient and payor.

## Conclusions

This case report demonstrates the potential power of dietary intervention, whether as a primary or adjunctive therapy in the current treatment armamentarium. The SCD intervention described here successfully induced endoscopic, radiologic, and histologic healing in an adult patient with moderate-to-severe Crohn’s disease over a 42-month period. This case report emphasizes the need for more systemic research on the role of diet in inflammatory bowel disease, improving clinical symptoms, reducing inflammatory burden, supporting mucosal healing, and influencing overall healthcare economics.
